# Consumption of betel quid contributes to sensorineural hearing impairment through arecoline-induced oxidative stress

**DOI:** 10.1038/s41598-019-49815-5

**Published:** 2019-10-10

**Authors:** Yen-Hui Chan, Tien-Chen Liu, Chun-Kang Liao, Yen-Fu Cheng, Ching-Hui Tsai, Ying-Chang Lu, Chin-Ju Hu, Hung-Ju Lin, Yungling Leo Lee, Chen-Chi Wu, Chuan-Jen Hsu

**Affiliations:** 1 Department of Otolaryngology, National Taiwan University Hospital, Taipei, Taiwan; 2 Department of Otolaryngology, Taichung Tzu Chi Hospital, Buddhist Tzu Chi Medical Foundation, Taichung, Taiwan; 3 Department of Medical Research, Taipei Veterans General Hospital, Taipei, Taiwan; 4 Department of Otolaryngology-Head and Neck Surgery, Taipei Veterans General Hospital, Taipei, Taiwan; 5 School of Medicine, National Yang-Ming University, Taipei, Taiwan; 6 Institute of Epidemiology and Preventive Medicine, College of Public Health, National Taiwan University, Taipei, Taiwan; 7 Health Management Center, National Taiwan University Hospital, Taipei, Taiwan; 8 Institute of Biomedical Sciences, Academia Sinica, Taipei, Taiwan; 9 Department of Medical Genetics, National Taiwan University Hospital, Taipei, Taiwan

**Keywords:** Peripheral neuropathies, Experimental models of disease, Risk factors

## Abstract

Betel quid is one of the most widely used psychoactive substances, and is consumed by approximately 10% of the world’s population. In addition to its carcinogenicity, betel quid has also been reported to affect many organs, including the brain, heart, lungs, gastrointestinal tract, and reproductive organs. As betel quid contains several neurotoxic ingredients, we hypothesize that it also possesses ototoxicity and may lead to sensorineural hearing impairment (SNHI). In this study, we investigated the contribution of betel quid consumption to SNHI in a large clinical cohort, and validated the pathogenetic mechanisms in *ex vivo* tissue explants. We enrolled a total of 2364 volunteers, and determined their audiologic results based on Z-scores converted from their original frequency-specific hearing thresholds. Using generalized linear regression, we identified a positive correlation between betel quid consumption and the Z-scores across different frequencies. Subsequently, we explored the toxicity of arecoline, the main neuroactive component of betel quid, on tissue explants from murine cochleae. Arecoline reduced cell activity in the explant cultures and induced apoptosis in the hair cells, probably through the effects of oxidative stress. These findings have expanded the potential hazards of betel quid to common neurological disorders, and provide insights into preventive strategies against SNHI caused by neurotoxic substances.

## Introduction

Sensorineural hearing impairment (SNHI) is the most common sensory disorder in humans. According to the report of the World Health Organization, it is estimated that approximately 466 million people worldwide have disabling hearing loss greater than 40 dB, and 34 millions of these are children (http://www.who.int/en/news-room/fact-sheets/detail/deafness-and-hearing-loss). SNHI in children is caused predominantly by genetic mutations^1^. By contrast, SNHI in adults, similar to other neurodegenerative disorders, involves an interplay between genetic and environmental factors^2–4^. In addition to noise exposure^5^, a number of toxic substances have been documented to contribute to SNHI, including ototoxic antibiotics, diuretics, chemotherapeutic agents, and tobacco^3,6^. In mammals, including humans, the damages result in permanent hearing loss due to the quiescence nature of the sensory epithelium of the cochlea^7,8^.

Betel quid or areca nut (the primary ingredient in betel quid) is among the most widely used psychoactive substances worldwide, along with tobacco, alcohol, and caffeine. Globally, more than 600 million people are estimated to consume betel quid or areca nut in some form^9–12^. Consumption is especially prevalent in the Asia-pacific region, including South and Southeast Asia, tropical Pacific, east Africa, as well as in emigrant communities arising therefrom. Both betel quid and areca nut are classified as carcinogenic to human beings by the International Agency for Cancer Research, and have been confirmed as important risk factors for oral, pharyngeal, and esophageal cancers^13^. In addition, the consumption of betel quid has also been related to various disorders, including myocardial infarction^14^, cardiac arrhythmia^15^, hepatotoxicity^16^, asthma^17^, diabetes^18^, metabolic syndrome^19^, premature delivery^20^, and infertility^21^. There is growing evidence that the predominant psychoactive agent in betel quid, arecoline, plays a critical role in the carcinogenesis process^22–24^.

Interestingly, experiments in cell lines revealed that arecoline of the betel quid causes neurotoxicity by enhancing oxidative stress and suppressing the antioxidant protective systems^25^. Despite these findings in cell lines, the real contribution of betel quid consumption to neurological disorders (such as SNHI) in humans remained unexplored. Accordingly, we investigated the effects of betel quid consumption on SNHI in a large clinical cohort, and then validated the pathogenetic mechanisms in *ex vivo* tissue explants.

## Materials and Methods

### Subject recruitment

From 2007 to 2017, Han Chinese volunteers aged between 40–80 years were prospectively recruited during routine health examination from the Health Management Center of National Taiwan University Hospital to establish a cohort for epidemiological and pathogenetic research on SNHI^26–28^. The subjects were interviewed by trained assistants with the use of a structured questionnaire detailing demographic data and family history of hearing loss. The questionnaire also served to obtain information on medical history including betel quid consumption, smoking, alcohol consumption, and systemic diseases, such as coronary artery disease, hypertension, diabetes, hyperlipidemia, and chronic hepatitis. Height and body weight were measured after an overnight fast to determine the body mass index (BMI).

All subjects and/or their parents provided informed consent before testing, and all procedures were approved by the Research Ethics Committees of National Taiwan University Hospital. All methods were performed in accordance with the relevant guidelines and regulations.

### Audiologic evaluations

All subjects underwent an otoscopic examination by an otologist to inspect ear pathology, and the audiologic results were assessed in terms of air- and bone-conduction thresholds of pure-tones using an audiometer (Grason-Stadler Inc., GSI 10). Air-conduction thresholds were measured at 0.25, 0.5, 1, 2, 4, and 8 kHz; and bone-conduction thresholds were measured at 0.5, 1, 2, and 4 kHz. Subjects with asymmetric sensorineural hearing loss, conductive hearing loss, and a 4-kHz dip on an audiogram were excluded since these audiologic features indicate the presence of other pathologies. Subjects with a history of any of the following were also excluded: external- or middle-ear diseases, hearing loss before the age of 30 years, high environmental noise exposure, exposure to ototoxic medication, brain tumor, vertigo, chronic renal failure under dialysis, head and neck radiation exposure, heavy smoking, alcoholism, or substance abuse.

Frequency-specific thresholds were converted to sex- and age-independent Z-scores, which represent the number of standard deviations by which the hearing threshold differs from the median at a given frequency^29^. Z-scores for each frequency were denoted as Z_250_, Z_500_, Z_1000_, Z_2000_, Z_4000_, and Z_8000_. Corresponding to the hearing levels at the low-tone, mid-tone, and high-tone frequencies, Z_250_ and Z_500_ scores were averaged as Z_low_, Z_1000_ and Z_2000_ scores as Z_mid_, and Z_4000_ and Z_8000_ as Z_high_^26^. Only Z-scores of the better hearing ear were used for subsequent analyses.

### Generalized linear regression

To investigate the associations between risk factors and Z-scores, we used the generalized linear regression model (GLM). All of the models were adjusted by age and gender. Coefficients (β) of all the risk factors and the 95% confidence intervals (CI) were estimated using the SAS Software version 9.1 (SAS Institute). Statistical significance was set at p < 0.05 based on two-sided estimation.

### Preparation of cochlear explants

**Figure 1:**
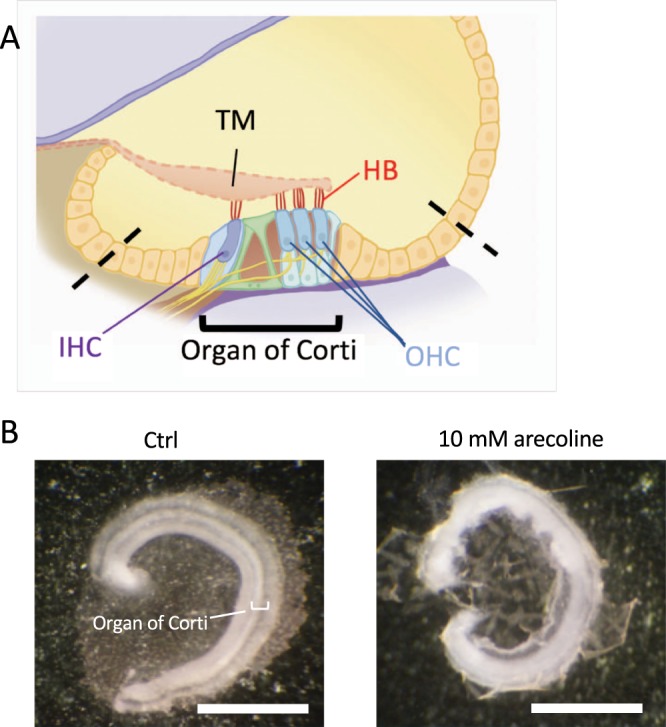
The effects of arecoline on cochlear explants. (**A**) The sketch of the cross section of the cochlear duct and the organ of Corti within it. Cochlear explants used in this study contained the region between the dashed lines with the removal of the tectorial membrane (TM). (**B**) The morphology of cochlear explants under stereo microscopy after 3 days of culture without and with 10 mM arecoline. HB, hair bundles; IHC, inner hair cells; OHC, outer hair cells; TM, tectorial membrane. Bar = 1 mm.

*Ex vivo* experiments were carried out by culturing cochlear explants containing the organ of Corti and spiral ganglions harvested from 3–5-day-old C57BL/6 mice (Fig. 1A)^26^. The explants dissected from the otic capsule were oriented with the hair cell-side up, attached to the Matrigel (Corning) coated on cell culture dishes (Greiner Labortechnik) in the Dulbecco’s Modified Eagle Medium (DMEM) (GIBCO) with 7% fetal bovine serum, and then kept at 37 °C in a humidified incubator under 5% CO_2_. To explore the toxic effects of arecoline on the cochlear explants, we used gradient concentrations, 200 μM, 800 μM, 2 mM, and 10 mM of arecoline, and examined the tissue morphology, hair cell arrangement, cell activity, and cytotoxicity. Rhodamine-phalloidin (Invitrogen) was used to label the f-actin to examine the cell morphology and the hair bundles of the hair cells. The cells were counterstained with 4′,6-diamidino-2-phenylindole (DAPI). All animal experiments were carried out in accordance with animal welfare guidelines and approved by the Institutional Animal Care and Use Committee (IACUC) of National Taiwan University College of Medicine.

### Cell activity and cytotoxicity

The cell activity of cochlear explants was investigated using the alamarBlue assay, which measures the reducing power of living cells quantitatively^30^. The alamarBlue reagent (10% v/v) was added into the culture medium and incubated for 24 h, and the culture medium was then transferred into 96-well plates. Subsequently, the fluorescent signals were examined by an ELISA reader using a fluorescence excitation wavelength of 570 nm and measuring emission at 585 nm.

The lactate dehydrogenase (LDH) Cytotoxicity Assay Kit (Pierce) was used to determine the amount of LDH released from the damaged cells^31^. The culture medium of the cochlear explants incubated for 24 h was collected at the indicated time points, and mixed with the LDH reaction mixtures at room temperature for 30 min. The reaction was terminated by adding the stop solution, and subsequently the absorbance was read by an ELISA reader at 490 nm and 690 nm. The absorbance values were processed according to the manufacturer’s instructions to calculate the LDH values.

### TUNEL assay

The terminal deoxynucleotidyl transferase dUTP nick end labeling (TUNEL) assay was performed by using the APO-BrdU TUNEL Assay Kit (Invitrogen). It is based on the binding of 5-bromo-2′-deoxyuridine 5′-triphosphate (BrdUTP) by the terminal deoxynucleotidyl transferase (TdT) at the 3′ hydroxyl termini of fragmented DNA^32^. This was followed by detecting BrdU with an anti-BrdU antibody using standard immunofluorescent techniques. After rinsing with ice cold phosphate-buffered saline (PBS) twice, the cochlear explants were permeabilized with 0.1% Triton X-100 in PBS. After washing, the explants were incubated in the TUNEL reaction mix for 1 h at 37 °C in the dark, and then labeled using the Alexa Fluor 488 dye-conjugated anti-BrdU antibody. Hair cells were stained by the phalloidin–rhodamine probe (Invitrogen) and then counterstained with DAPI. The cochlear explants were mounted and photographed using a confocal microscope (Zeiss LSM 780). The apical, middle, and basal parts of each explant were scanned for comparison.

### Cellular oxidative stress

Reactive oxygen species (ROS) within the cochlear explants were examined using the CellROX Green fluorogenic probe (Invitrogen)^33^. At 30 min before the indicated time points, CellROX Green Reagent was added to the cochlear explants at a final concentration of 5 μM and incubated at 37 °C in a 5% CO_2_ humidified incubator for 30 min. Then the medium was removed, and the cochlear explants were washed 3 times with ice cold PBS, fixed with 4% paraformaldehyde in PBS, and permeabilized with 0.5% Triton X-100. The hair bundles were labeled with phalloidin and the cells were counterstained with DAPI. The stained cochlear explants were then examined by Zeiss LSM 780 laser scanning confocal microscopy immediately. The apical, middle, and basal parts of each cochlear explant were scanned for comparison. The images were processed by ImageJ (NIH, Bethesda, MD, USA).

## Results

### Betel quid consumption contributed to SNHI in humans

In total, 2364 subjects, including 1306 men and 1058 women, with a mean age of 54.0 ± 7.7 years, were included in the analyses. The basic characteristics of these subjects are shown in Table 1. Fifty-five (2.3%) of the 2364 subjects consumed betel quid.

**Table 1:** Basic characteristics of the 2364 subjects.

Variables	Value*
Age (years)	54.0 ± 7.7
Male sex	1306 (55.1)
Coronary heart disease	66 (2.8)
Hypertension	398 (16.8)
Diabetes	136 (5.8)
Hyperlipidemia	123 (5.2)
Chronic hepatitis	129 (5.5)
BMI	24.0 ± 3.2
**Smoking**	
No	2064 (87.3)
Yes	300 (12.7)
**Alcohol consumption**	
No	1670 (70.6)
Yes	694 (29.4)
**Betel quid consumption**	
No	2309 (97.7)
Yes	55 (2.3)

*mean ± S.D. or n (%).

Under the generalized linear regression model, correlations with statistical significance were identified between a number of risk factors and the Z scores (Table 2), including the positive correlation between smoking and the Z_mid_ (95% CI 0.01~0.15, *p* = 0.03) and Z_high_ (95% CI 0.10~0.36, *p* = 0.0004) scores, as well as the positive correlation between the alcohol consumption and the Z_high_ score (95% CI 0.01~0.20, *p* = 0.02). The consumption of betel quid correlated positively with the Z_low_ (95% CI 0.08~0.34, *p* = 0.002), Z_mid_ (95% CI 0.16~0.47, *p* < 0.0001), and Z_high_ (95% CI 0.40~0.94, *p* < 0.0001) scores, indicating that betel quid consumption contributed to elevated hearing thresholds across different frequencies in humans.

**Table 2:** Generalized linear regression analyses of all risk factors affecting Z scores.

	Z_low_			Z_mid_			Z_high_		
	β	95% CI	p value	β	95% CI	p value	β	95% CI	p value
Coronary heart disease	0.02	(−0.10, 0.14)	0.73	0.04	(−0.10, 0.18)	0.54	0.01	(−0.24, 0.26148)	0.93
Hypertension	−0.05	(−0.11, 0.003)	0.07	−0.05	(−0.11, 0.01)	0.10	−0.14	(−0.25, −0.03)	0.01
Diabetes	0.10	(0.01, 0.19)	0.02	0.15	(0.05, 0.25)	0.003	0.25	(0.07, 0.43)	0.01
Hyperlipidemia	−0.01	(−0.10, 0.08)	0.75	−0.02	(−0.12, 0.08)	0.68	0.01	(−0.17, 0.20)	0.90
Chronic hepatitis	−0.03	(−0.11, 0.06)	0.57	0.002	(−0.10, 0.10)	0.95	0.11	(−0.07, 0.29)	0.21
BMI	0.01	(−0.001, 0.01)	0.10	0.01	(0.01, 0.02)	0.0002	0.02	(0.004, 0.03)	0.01
Smoking	0.05	(−0.01, 0.11)	0.14	0.08	(0.01, 0.15)	0.03	0.23	(0.10, 0.36)	0.0004
Alcohol consumption	−0.01	(−0.06, 0.03)	0.62	−0.01	(−0.06, 0.05)	0.78	0.11	(0.01, 0.21)	0.02
Betel quid consumption	0.21	(0.08, 0.34)	0.002	0.31	(0.16, 0.47)	<0.0001	0.67	(0.40, 0.94)	<0.0001

Consistent with our previous studies that demonstrated a significant association between obesity and SNHI^26,27^, BMI correlated positively with the Z_mid_ (95% CI 0.01~0.02, *p* = 0.0002) and Z_high_ (95% CI 0.004~0.03, *p* = 0.01) scores. Of note, diabetes and betel quid consumption were the two factors that were significantly associated with all the Z_low_, Z_mid_, and Z_high_ scores.

### Arecoline caused derangement of hair cells in the organ of Corti

**Figure 2:**
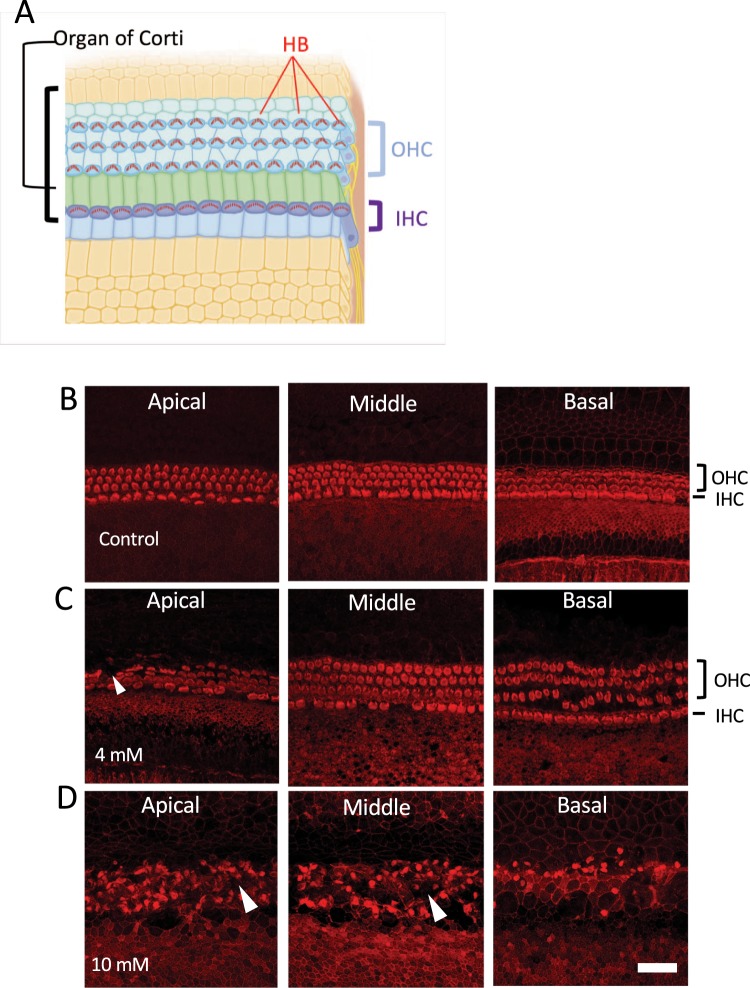
The effects of arecoline on cochlear hair cells. (**A**) A schematic diagram showing the orientation of both outer hair cells (OHCs) and inner hair cells (IHCs). The mechanosensitive hair bundles (HB) of hair cells are clusters of stereocilia on top of each hair cell and can be stained with phalloidin. (**B**–**D**) The hair bundles labeled by phalloidin after 30 minutes of arecoline treatment. In the control group (**B**), the three rows of outer hair cells and one row of inner hair cells retained the original regular arrangement and v-shaped hair bundles. By contrast, explants treated with 4 mM (**C**) or 10 mM (**D**) arecoline showed disrupted hair cell arrangement with disappearance of some hair bundles, resulting in vacancies in the hair cell regions (arrowheads). HB, hair bundles; IHC, inner hair cells; OHC, outer hair cells. Bar = 10 μm (**B**–**D**).

In this study we focused on the organ of Corti region to evaluate the effects of arecoline. Figure 1A shows the cross-section of the cochlear duct and the organ of Corti, which is composed of many types of cells, and the most important are the hair cells with hair bundles (HB) on the apical membrane. In contrast to untreated controls which showed a striped pattern with translucent bands at the organ of Corti (Fig. 1B, left panel), cochlear explants treated with arecoline revealed an opaque appearance (Fig. 1B, right panel). In the untreated cochlear explants, three rows of outer hair cells and one row of inner hair cells retained the original regular arrangement and the v-shaped hair bundles (Fig. 2A,B). On the contrary, the cochlear explants with the short-term and high-dose treatment of 4 mM or 10 mM arecoline showed disrupted hair cell arrangement with the disappearance of some hair bundles, resulting in gaps in the hair cell regions, which phenomena is not caused by the long-period culture (Fig. 2C,D, arrowheads).

### Arecoline decreased cell activity and exerted cytotoxicity in cochlear explants

**Figure 3:**
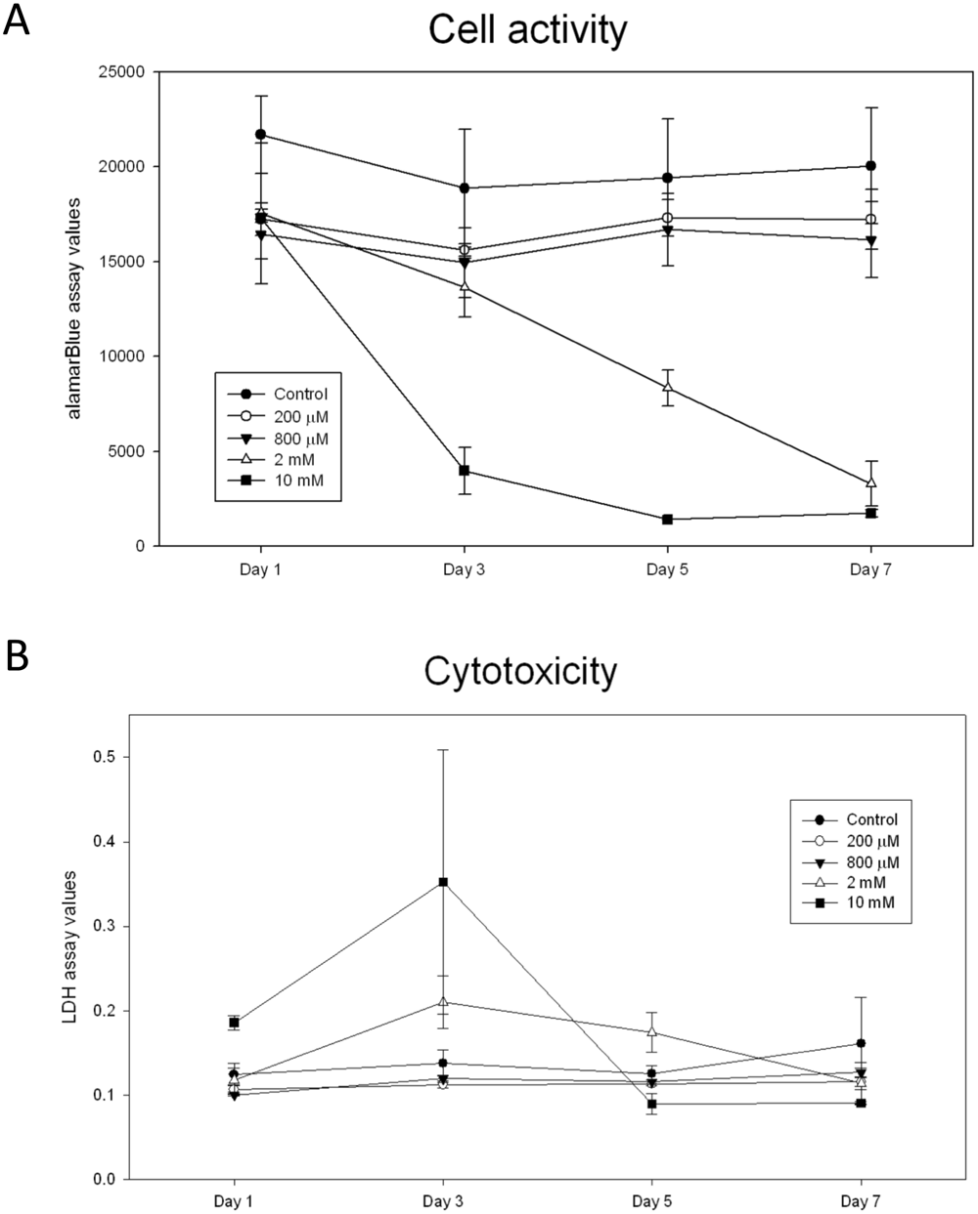
The cell activity and cytotoxicity assays of the cochlear explants. (**A**) The cell activity determined by the alamarBlue^®^ assay. Experiments were performed under 5 different conditions: control, 200 μM, 800 μM, 2 mM, and 10 mM arecoline (n = 3). The data were recorded on day 1, 3, 5, and 7. (**B**) The cytotoxicity determined by LDH values. Experiments were also performed under 5 different conditions: control, 200 μM, 800 μM, 2 mM, and 10 mM arecoline. The data were recorded on day 1, 3, 5, and 7 (n = 3). Data are expressed as mean ± SEM.

After treatment with 10 mM arecoline, the cell activity of cochlear explants decreased dramatically on day 3 and stayed at a low level until day 7, indicating that cells were seriously damaged within 3 days of treatment (Fig. 3A). A rather moderate decline in the cell activity was observed after treatment with 2 mM arecoline, which showed similar cell activity level on day 3 as the cochlear explants treated with 800 μM or 200 μM arecoline, but started to decline on day 5, and finally diminished to the same level as the 10 mM arecoline-treated cochlear explants on day 7. This finding suggested that 2 mM of arecoline is sufficient to cause toxic effects in cochlear explants.

Figure 3B illustrates the cytotoxicity in terms of LDH levels after treatment with various concentrations of arecoline. Parallel to the results in the cell activity, treatment with 200 μM or 800 μM arecoline did not cause obvious cell death in the cochlear explants, whereas treatment with 2 mM or 10 mM arecoline led to a surge in LDH on day 3, concurrent with the decrease in cell activity shown in Fig. 3A. The LDH levels then decreased on day 5 commensurate to that of the control group, indicating that the cell death caused by arecoline mainly occurred within the first 3 days.

### Arecoline induced cell death in cochlea explants

**Figure 4:**
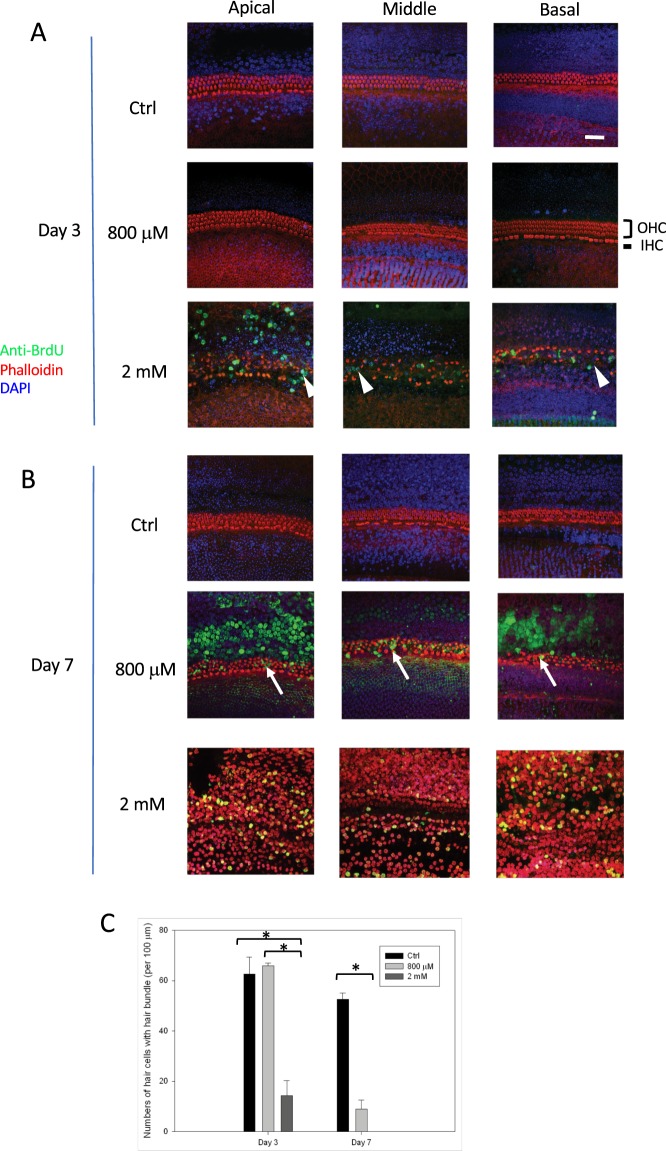
The cell death detected by the TUNEL assay. The BrdU labeled the nuclei of dead cells (green) on day 3 (**A**) and day 7 (**B**). Red: phalloidin-labeled f-actin and hair bundles; blue: DAPI-labeled nuclei. On day 3 after treatment with 2 mM arecoline, positive green TUNEL signals appeared mainly within the hair cells (arrowheads), which then progressed to a severely damaged pattern of a wide-spread fragmented nuclei on day 7. After treatment with 800 μM arecoline, there were no positive green TUNEL signals on day 3, but some apoptotic cells (arrows) within the hair cell region could be observed on day 7. (**C**) The numbers of hair cells with distinguishable hair bundles were counted per 100 μm section of the middle region of cochlear duct. At day 3, the 2 mM arecoline-treated explants retained significantly fewer hair cells with distinguishable hair bundles than the untreated and 800 μM arecoline-treated explants, which further decreased to 0 cells at day 7. At day 7, the 800 μM arecoline-treated explants also revealed significantly fewer hair cells with distinguishable hair bundles than the untreated explants. Error bars represent SEM. HC, hair cells; IHC, inner hair cells; OHC: outer hair cells. Bar = 10 μm (**A**,**B**). *p < 0.01.

To specify the cell types where death events occurred after arecoline treatment, we performed the TUNEL assay to label the DNA breaks of the apoptotic cells. On day 3, compared to the relatively normal appearance observed in the controls and cochlear explants treated with 800 μM arecoline, the cochlear explants treated with 2 mM arecoline showed positive nuclear BrdU stains at the organ of Corti (Fig. 4A, lower panel, arrowheads).

On day 7, cochlear explants treated with 800 μM arecoline revealed some degeneration of the hair bundles accompanied with some positive BrdU stains at the organ of Corti region (Fig. 4B, middle panel, arrows), whereas the cochlear explants treated with 2 mM arecoline exhibited a severely injured pattern, where phalloidin stains showed no discernible hair bundles and demonstrated an apoptotic pattern, accompanied by scattered BrdU stains (Fig. 4B, lower panel). These findings indicated that apoptosis after arecoline treatment developed predominantly in the hair cells. The numbers of hair cells with distinguishable hair bundles were counted per 100 μm section of cochlear duct (Fig. 4C). At day 3, the 2 mM arecoline-treated cochlear explants retained significantly fewer hair cells with distinguishable hair bundles (n = 14 ± 6) than the untreated (n = 62 ± 7) and 800 μM arecoline-treated cochlear explants (n = 66 ± 1) (both p < 0.01), which further decreased to 0 cells at day 7. At day 7, the 800 μM arecoline-treated cochlear explants also revealed significantly fewer hair cells with distinguishable hair bundles (n = 9 ± 4) than the untreated cochlear explants (n = 52 ± 3).

### Arecoline induced ROS generation in the cochlea

**Figure 5:**
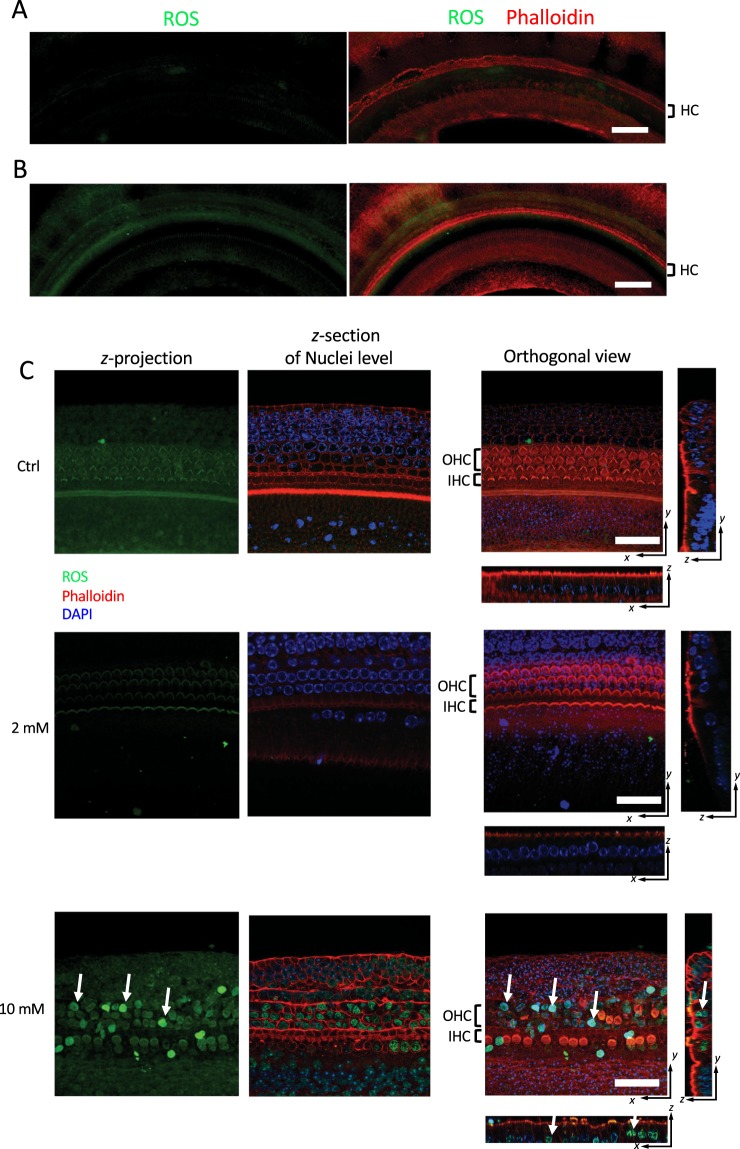
ROS generated in the cochlear explant. Cellular oxidative stress within the cochlea explant was examined using a fluorogenic probe which could be oxidized by reactive oxygen species (ROS). On day 2, there were no detectable ROS signals (green) in the untreated explants (**A**), while fuzzy fluorescent signals were detected around the hair cell band region (marked as HC region) in the 2 mM arecoline-treated explants (**B**). (**C**) The ROS signals could be detected within 3 h when the arecoline concentration was increased to 10 mM, especially in the hair cells (arrows). Disruption and disappearance of hair bundles within the hair cell region could also be observed. The z-projection column demonstrates the summed-up signals of all the optical sections, which represents the total ROS signal in the explant. The z-section column is specifically for the section at the nuclei level. The images of 10 mM group showed that many ROS signals were detected at the nuclei, indicating the oxidative stress generated (C, lower panel). HC, hair cells; IHC, inner hair cells; OHC: outer hair cells. Bar = 80 μm (**A**,**B**) and 30 μm (**C**).

On day 2, there were detectable ROS signals (green) in the untreated cochlear explants (Fig. 5A), while fuzzy fluorescent signals for ROS were detected around the hair cell band region in the 2 mM arecoline-treated cochlear explants (Fig. 5B). With the arecoline concentration being increased to 10 mM, ROS signals could be detected as early as 3 h after treatment, especially at the nuclei of the hair cells (Fig. 5C, arrows). It is noteworthy that there were some weak signals could be detected at the hair bundles, but we only focused on the nucleus-shaping signal in this study. Meanwhile, derangement and disappearance of hair bundles were also observed.

## Discussion

In this study, we observed that the consumption of betel quid constituted a significant risk factor for SNHI in a large clinical cohort. To elucidate the underlying mechanisms, we then investigated the toxicity of arecoline, the main neuroactive component of the betel quid, on tissue explants harvested from murine cochleae. Our findings revealed that arecoline could reduce the cell activity and induce apoptosis in the cochlear explants, and confirmed oxidative stress as one of the possible mechanisms.

The cochlea is a highly differentiated and structured organ containing multiple types of cells, including inner hair cells, outer hair cells, supporting cells, spiral ganglion neuronal cells, as well as other specialized epithelial cells. The auditory function and physiological homeostasis are maintained by intricate interactions between these specialized cells. As such, even though several cell lines are available for hearing research^34^, organotypic cultures remained an invaluable experimental model. In this study, we dissected and cultured the whole cochlear explants from mice to simulate the effects of arecoline in the *in vivo* condition. Our findings revealed that arecoline started to exhibit cytotoxic effects on the cochlea explants at a concentration between 800 μM and 2 mM (Fig. 3A,B). Notably, this cytotoxic concentration is higher than that of 50–200 μM in the neural cell lines^25^, indicating that cochlear explants are more resistant to arecoline toxicity than neural cell lines. It has been reported that the supporting cells in the cochlea could protect hair cells by secreting Heat shock protein 70 (HSP70)^35^. Therefore, it is conceivable that the intact organotypic structure of the cochlear explant might provide the hair cells with additional protective effects against toxic agents like arecoline.

Next, we conducted multi-parameter cell viability and cytotoxicity assessments to delineate the profile of tissue adaptability in response to arecoline treatment. The alamarBlue assay measures the metabolic activity of the mitochondrial enzymes of living cells (Fig. 3A), whereas the LDH assay detects the lactate dehydrogenase released from the cytosol of lysed cells^36^ (Fig. 3B). As the surge of LDH on day 3 after arecoline treatment represented the destruction of the integrity of the cytoplasmic membrane, it could be inferred that arecoline caused irreversible changes in the cells of the cochlear explants, resulting in decreased cellular metabolic activity, and eventually cell death.

To specify the types of cells damaged by the arecoline treatment, we further performed TUNEL assays to investigate the apoptotic process. Our findings showed that, corresponding to the morphological changes such as hair bundle degeneration, apoptosis mainly occurred in the hair cell region of the cochlear explants (Fig. 4). Different ototoxic substances could cause specific damage to different sub-populations of cells in the cochlea. For instance, ouabain leads to degeneration of neuronal cells in the spiral ganglion^37^, erythromycin causes dysfunction in the epithelial cells of the stria vascularis^38^, aminoglycosides induce death of the outer and inner hair cells^39,40^, whereas platinum-containing chemotherapeutic agents result in a wider range of damage involving hair cells, neuronal cells, and epithelial cells of the stria vascularis^41^. As our findings revealed that the hair cells were the predominant cell type damaged by arecoline, it could be assumed that the ototoxic mechanisms of arecoline might resemble those of the aminoglycosides and platinum-containing chemotherapeutic agents. Previous studies demonstrated that overproduction of ROS might play a pivotal role in the ototoxic mechanisms of aminoglycosides^39^ and platinum-containing chemotherapeutic agents^41^; and oxidative stress has also been implicated in the carcinogenesis related to betel quid chewing^42^.

The postulation regarding the ototoxic mechanisms of arecoline was confirmed by our observation that strong ROS signals were detected in the hair cell regions of the cochlear explants after arecoline treatment (Fig. 5B,C). This finding of increased ROS levels in the cochlear explants is in agreement with previous studies in neural cell lines which reported that arecoline might induce neurotoxicity through increased oxidative stress^25,43^. Elevated ROS generation and subsequent apoptosis have been implicated in various types of hearing loss, including noise-induced hearing loss, ototoxicity-induced hearing loss, and age-related hearing impairment^4,44,45^. In a healthy cell, a delicate balance of pro- and anti-apoptotic factors exists, allowing it to live and proliferate. In stress situations like noise exposure, exposure to ototoxic drugs, and aging, this balance might be disturbed, and specific cells in the cochlea may enter the apoptotic death program. The knowledge concerning the pathogenetic roles of ROS and apoptosis have advanced the preventive strategies against hearing loss. For example, the application of certain antioxidant molecules, such as Q-ter^46^, N-acetylcysteine^47,48^, D-methionine^49–51^, and glutathione^52^, has been demonstrated to be effective in preventing the development of noise-induced and ototoxicity-induced hearing loss.

In the hair cell region, the outer hair cells appeared to be more vulnerable to the arecoline treatment than the inner hair cells. Previous studies revealed that outer hair cells were susceptible to various apoptosis-inducing stimuli, and significant apoptosis in outer hair cells was observed in noise-induced hearing loss^53^, as well as in hearing loss caused by aminoglycosides^40,54^ and cisplatin^55^. Different subpopulations of cells in the cochlea might exhibit different expression patterns of ROS-related proteins. For instance, the superoxide-producing NADPH oxidase 3 (NOX3) is highly expressed in the hair cells of the organ of Corti^56^, contributing to differences in the ROS concentration among different cochlear cells. In addition, the differences in the expression of certain apoptosis-regulating molecules (e.g. Bcl2 and Bcl6) between inner and outer hair cells, as shown in recent transcriptomic studies^57,58^, might also be a possible explanation for the susceptibility of outer hair cells to early cell death.

To our knowledge, this study is amongst the first to document the relevance of betel quid consumption to common neurological diseases such as SNHI. However, some limitations of this study deserve discussion. First, the cytotoxic concentrations of arecoline determined in the cochlear explants were not validated *in vivo*, because it was impossible to obtain cochlear tissue or fluid from the human subjects. However, as our findings have confirmed that arecoline resulted in increased ROS in the hair cells, it would not be surprising that long-term accumulation of oxidative stress might cause damage to the inner ear in humans and contribute to SNHI in individuals who consume betel quid. Second, although our results showed that increased ROS could be the crucial factor which induced hair cell apoptosis after arecoline treatment, it must be emphasized that there might be other apoptosis-inducing stimuli which do not depend on ROS production that could also contribute to the apoptotic process. It has been reported that increased Ca^2+^ in the outer hair cells^59^ or the activation of calcineurin^60^ may also trigger apoptotic pathways without ROS production. Further studies are warranted to elucidate the comprehensive mechanisms of arecoline in the pathogenesis of SNHI.

In conclusion, we identified that betel quid consumption might contribute to SNHI in humans, and confirmed that arecoline of the betel quid could induce apoptosis in hair cells through oxidative stress in *ex vivo* experiments. These findings have important public health implications by expanding the potential hazards of betel quid to common neurological disorders, and provide insights into the preventive strategies against SNHI or other neurological diseases caused by environmental neuro-toxic substances.
